# 12 tips for developing Educational Conferences for Physicians

**DOI:** 10.12688/mep.19901.2

**Published:** 2024-04-30

**Authors:** Kelly McCoy, Lisa Fore-Arcand

**Affiliations:** 1 Continuing Medical Education, Eastern Virginia Medical School, Norfolk, Virginia, 23507, USA; 2 Department of Psychiatry & Behavioral Sciences, Eastern Virginia Medical School, Norfolk, Virginia, 23507, USA

**Keywords:** Physician Education, CME, Curriculum Development

## Abstract

The education of a physician is a life-long process that starts in medical school and extends throughout their career. Healthcare is a dynamic field characterized by continuous advancements in medicine, evolving treatment options, changing regulations, care models, and technology. Physicians must keep up-to-date with new practices, procedures, medications, and diseases and fulfill the educational requirements to maintain their medical licensure. Continuing education for physicians serves the essential purpose of nurturing lifelong learning, ensuring that medical practices align with the latest standards, and ultimately enhancing the quality of patient care and outcomes. In a broader context, physician education encompasses all activities designed to enhance skills, professional performance, and relationships that physicians employ to provide services to patients and the public and to improve collaborations within the field. This paper outlines a step-by-step plan for designing high-quality educational conferences for practicing physicians in any field. It aims to assist in developing ongoing education, aligning physician practices with the latest medical care standards, and optimizing their clinical performance to improve patient and community health.

## Introduction

Throughout a physician’s career, healthcare is continuously changing ( Thompson, 2014). With advances and updates in medicine, treatment options, guidelines and regulations, models of care, and technology, physicians must stay abreast of these changes, not only for the care and safety of their patients, but to maintain licensure and certifications. ( HealthStream, 2021). This can be accomplished through physician continuing education, which facilitates lifelong learning to enhance their skills, knowledge, and performance, build stronger interdisciplinary relationships, and increase patient communications and outcomes ( VanNieuwenborg *et al.*, 2016).

Following are 12 tips for developing strong medical education conferences to ensure that physicians are current with medical knowledge and obtain the credits necessary to maintain their medical licensure. This guide was developed by physician educators with more than 30 combined years of medical education experience, and is based on office procedures and accreditation compliance, to assist planners of medical education and professional development activities. The steps do not need occur in this specific order and should serve as a guide to developing physician educational activities.

## Tip 1

### Design the gap analysis

When developing physician education, it is essential to identify the problem or professional practice gap to be addressed. The difference between current and ideal performance and/or outcomes is known as the professional practice gap. The problem can address anything in healthcare that relates to a physician’s knowledge, competence, or performance, to patient outcomes, or non-clinical issues such as wellness, coding/billing, and faculty development. Once the problem is identified, determine the ideal performance and/or outcome ( Thomas *et al*., 2016). To do this, ask what the physician needs to learn or do differently to close the gap. Once this step is complete, you can craft the learning objectives. When doing so, ask what will the learner be able to do as a result of attending the activity. These are learning objectives that link to the educational need and should be specific, measurable, achievable, relevant and timebound (SMART) ( Orr *et al*., 2022). Using the information gathered, identify the desired results that reflect how the learner can implement the education/information into clinical practice. Table 1 shows an example of a planning tool used to collect and plan an educational activity.

**Table 1.** An example of a planning tool used to collect and plan an educational activity.

Professional Practice Gap	Educational Needs	Designed to Change	Learning Objectives	Desired Results
**Example:** The number of older people with HIV is increasing to include those younger adults who were perinatally infected. This aging population is experiencing an increased burden of age associated comorbidities in an era where antiretroviral therapy is effective ( Sangarlangkarn *et al.*, 2021).	**Example:** Physicians may not be informed of updated guidelines in the treatment of geriatric patients with HIV (PWH). Physicians do not receiving enough training regarding the treatment and care of aging PWH. There are unique challenges and barriers for older PWH who receive care to include: sexual health, self- care, psychosocial and cognitive issues.	_X__ Learner Knowledge/ Competence _X__ Learner Performance ____ Patient Health ____ Community/Population Health	**Example:** ‘As a result of attending this activity, the learner should be able to...’ Within 60 days of completing this educational activity, the learner will . . . Recommend and implement at least two treatment options based on updated guidelines for geriatric PWH. Implement clinical and practice strategies incorporating current guidelines. Develop at least three strategies addressing the unique challenges and barriers faced by older PWH.	**Example:** (i.e. Improved knowledge, increased competence, increased performance, increased patient outcomes.) Improve selection and prescription of medications and treatment for aging HIV patients. Improve knowledge in the implementation of current guidelines regarding HIV patient care.

## Tip 2

### Choose a planning committee

Consider having a diverse and interprofessional representation of stakeholders interested in the education being developed. The individuals should provide expertise on choosing the best topics to cover and speakers to deliver the content. This group will provide feedback regarding the educational need, conference goals and learning objectives, and provide input regarding the budget and assessment ( Gross *et al*., 2013).

## Tip 3

### Define your target audience

Who should attend this activity? This is the target audience. The target audience can be as broad or as specific as you’d like, but should have a direct interest in the educational content. While physicians are your primary focus, additional audiences, such as advanced practice providers, nurses, and allied health professionals, should be welcomed with the understanding that the content was developed to meet the target audience’s needs. ( Bowser, 2015).

## Tip 4

### Decide what educational format is best suited to meet the educational goals

The method of delivery is essential to ensuring the success of the activity. Consider the generational gap of your audience and which teaching methodologies are best for achieving the key learning points of this activity. ( Biddle & Huffman, 1994; Bowser, 2015) For instance, online learning formats are optimal for developing educational conferences designed to reach a broader audience and adapt to the time constraints of physicians. If developing a conference to enhance skills, a simulated or hands-on environment might be the most appropriate method for learners to obtain hands-on instruction, practice techniques, and receive feedback.

## Tip 5

### Identify core competencies

Core competencies will help you categorize what type of change the learner can expect from attending the activity. Some examples of competencies are patient care, professionalism, interpersonal and communication, and quality improvement. For instance, after reviewing patient feedback, physicians have received low marks regarding their bedside manners. Plan an activity focused on interpersonal and communication core competencies. If the event addresses improving patient diagnosis, management and treatment, the activity will align with patient care ( Eno *et al.,* 2020).

## Tip 6

### Develop the agenda

Based on the gap analysis, learning objectives, and target audience, describe the content that should be covered to close the identified professional gap. The planning committee should choose topics that will meet the activity’s learning objectives. When building the agenda, remember to add time for question and answer sessions, breaks, meals, reflection, and evaluation ( Gross *et al*., 2013). For long, full-day conferences, vary the delivery method to maintain the learner’s interest. Content can be delivered by didactic lectures, panel discussions, simulations, and hands-on workshops.

## Tip 7

### Confirm faculty

To determine the most appropriate speaker to deliver the content, research experts familiar with the clinical practice issues and problems related to the field(s) being addressed. Choose speakers based on the specifically determined criteria that will fulfill the educational needs of the target audience. The potential speaker should be engaging and highly regarded by the medical community ( Muroff, 2005). It is essential to review their curriculum vitae, financial disclosures and, if available, any previously recorded lectures.

Within the formal invitation, communicate the target audience, the educational need, and the learning objectives to ensure the educational content is designed appropriately.

## Tip 8

### Assessing potential barriers

When developing educational activities, plan to address potential barriers and have alternative options. Expense and time have been identified as the most common barriers to obtaining continuing education ( Pott *et al.,* 2021). Other barriers to consider are staffing or institutional restrictions, accessibility, and convenience regarding time of day and/or location. Addressing barriers early in the planning will support developing education that accommodates learners of all abilities. A couple of examples of adapting to barriers would be pivoting to the design of online learning for physicians during the pandemic when in-person gatherings were prohibited and ensuring accessibility through services such as closed captioning (CC) or interpreters to accurately communicate content.

## Tip 9

### Create a budget

Consider the financial goal of the activity. List all possible expenses to help determine the registration fees and how much is needed from outside support, including educational grants and exhibitor fees ( Muroff, 2005). The following are just a few expenses to consider when developing the budget: honorarium, speaker travel, accreditation costs, venue fees, food and beverage, and marketing.

## Tip 10

### Promote the activity

Develop a marketing timeline and itemized budget to help stay on track. Analyze the demographics of the intended audience to help determine the promotional methods. Consider electronic or physical mailings, whether to purchase a mailing list, and what social media platforms to use, if any.

## Tip 11

### Evaluate and measure change

One objective of continuing education is to close the identified gap. It is essential to measure change in physician knowledge, competence, performance, and patient outcomes. There are several ways to measure change, pre- and post-tests, subjective evaluations, and evaluations with feedback are a few examples. Make sure you choose a method that best measures if the learning objectives and outcomes were achieved and how well, and when possible, provides feedback to the physician to reinforce their learning and to the presenters to evaluate their teaching skills ( Thomas *et al.*, 2016).

## Tip 12

### Debrief

A step that can often be forgotten, but is critical to developing future educational activities, is debriefing. Debriefing allows for reflection among the planning committee where they can discuss strengths, weaknesses, learner evaluation and feedback, and discuss future changes to improve the learning activity and/or outcomes. It is a vital step for improving performance and clinical outcomes. Debriefing is intended to boost critical thinking and advance future clinical practice and improve future educational activities ( Institute for Healthcare Improvement, 2021).

## Limitations and conclusion

It is critical to provide quality physician education that is dynamic and engaging to ensure that medical care provided worldwide reflects the most up-to-date information and training. Limitations to providing physician education can include time constraints, cost, industry influence, impact assessment, and limited interactivity ( Pott *et al*., 2021). Mitigating these limitations is imperative to providing quality unbiased education. Solutions can include on-line learning, restricting industry support, designing comprehensive post activity evaluations, and designing interactive learning formats. Planning and implementing quality continuing medical education can be challenging, but given the structure provided by these step-by-step tips, apprehension can be reduced. Physicians never stop learning and providing structured opportunities for them to learn can be rewarding. Continuing education facilitates life-long learning for physicians to provide the best medical care to their patients. Future planning for physician education might address changes to state and national guidelines, pharmaceutical updates, and quality and safety topics such as initiatives and best practices, and should consider accreditation compliance, when necessary.

## Data Availability

No data are associated with this article.
