# Supplementary material for: 12 tips for developing Educational Conferences for Physicians
Source: MedEdPublish (2016). 2024 Apr 30;14:3. Originally published 2024 Jan 9. [Version 2] doi: 10.12688/mep.19901.2 (PMC10940845; doi:10.12688/mep.19901.2)
Supplement: Peer review history

# Reviewer report: Reviewer response for version 2

**DOI:** 10.21956/mep.21824.r36731

**Published:** 2024-05-14

**Authors:** Peirce W. Johnston

**Affiliations:** 1 University of Cincinnati College of Medicine, Cincinnati, USA

I have no new comments.

 Thank you,

 Peirce Johnston MD

If evidence from practice is presented, are all the underlying source data available to ensure full reproducibility?

Not applicable

Is the topic of the practical tips discussed accurately in the context of the current literature

Yes

Are all factual statements correct and adequately supported by citations?

Yes

Are the conclusions drawn balanced and justified on the basis of the presented arguments?

Yes

Are arguments sufficiently supported by evidence from the published literature and/or the authors’ practice?

Yes

Reviewer Expertise:

Limited researcher but my areas are in Medical Student Education and PsychOncology

I confirm that I have read this submission and believe that I have an appropriate level of expertise to confirm that it is of an acceptable scientific standard.

# Reviewer report: Reviewer response for version 2

**DOI:** 10.21956/mep.21824.r36732

**Published:** 2024-05-09

**Authors:** Ritcha Saxena

**Affiliations:** 1 University of Minnesota, Duluth, Minnesota, USA

No further comments.

If evidence from practice is presented, are all the underlying source data available to ensure full reproducibility?

Not applicable

Is the topic of the practical tips discussed accurately in the context of the current literature

Yes

Are all factual statements correct and adequately supported by citations?

Partly

Are the conclusions drawn balanced and justified on the basis of the presented arguments?

Yes

Are arguments sufficiently supported by evidence from the published literature and/or the authors’ practice?

Partly

Reviewer Expertise:

Medical Education, AI, Technology-enhanced Education

I confirm that I have read this submission and believe that I have an appropriate level of expertise to confirm that it is of an acceptable scientific standard.

# Reviewer report: Reviewer response for version 2

**DOI:** 10.21956/mep.21824.r36730

**Published:** 2024-05-09

**Authors:** Rie Raffing

**Affiliations:** 1 The Parker Institute, Bispebjerg & Frederiksberg Hospital,, University of Copenhagen, Copenhagen, Denmark

Thank you for your response to the review.

 I do not have furhter comments.

If evidence from practice is presented, are all the underlying source data available to ensure full reproducibility?

Not applicable

Is the topic of the practical tips discussed accurately in the context of the current literature

Partly

Are all factual statements correct and adequately supported by citations?

No

Are the conclusions drawn balanced and justified on the basis of the presented arguments?

Yes

Are arguments sufficiently supported by evidence from the published literature and/or the authors’ practice?

Partly

Reviewer Expertise:

Medical education

I confirm that I have read this submission and believe that I have an appropriate level of expertise to confirm that it is of an acceptable scientific standard.

# Reviewer report: Reviewer response for version 2

**DOI:** 10.21956/mep.21824.r36734

**Published:** 2024-05-09

**Authors:** Anita Kablinger, Alexandra Toloczko

**Affiliations:** 1 Department of Psychiatry and Behavioral Medicine, Carilion Clinic, Roanoke, Virginia, USA; 2 Virginia Tech Carilion School of Medicine, Roanoke, VA, USA

No further comments

If evidence from practice is presented, are all the underlying source data available to ensure full reproducibility?

Not applicable

Is the topic of the practical tips discussed accurately in the context of the current literature

Partly

Are all factual statements correct and adequately supported by citations?

Partly

Are the conclusions drawn balanced and justified on the basis of the presented arguments?

Partly

Are arguments sufficiently supported by evidence from the published literature and/or the authors’ practice?

Partly

Reviewer Expertise:

Medical and research education.

We confirm that we have read this submission and believe that we have an appropriate level of expertise to confirm that it is of an acceptable scientific standard.

# Reviewer report: Reviewer response for version 2

**DOI:** 10.21956/mep.21824.r36733

**Published:** 2024-05-07

**Authors:** Anne M. Messman

**Affiliations:** 1 Department of Emergency Medicine, University Health Center-6G, Wayne State University School of Medicine, Detroit, MI, USA

No new comments.

If evidence from practice is presented, are all the underlying source data available to ensure full reproducibility?

Not applicable

Is the topic of the practical tips discussed accurately in the context of the current literature

Yes

Are all factual statements correct and adequately supported by citations?

Yes

Are the conclusions drawn balanced and justified on the basis of the presented arguments?

Yes

Are arguments sufficiently supported by evidence from the published literature and/or the authors’ practice?

Yes

Reviewer Expertise:

medical education

I confirm that I have read this submission and believe that I have an appropriate level of expertise to confirm that it is of an acceptable scientific standard.

# Reviewer report: Reviewer response for version 1

**DOI:** 10.21956/mep.21317.r36110

**Published:** 2024-03-25

**Authors:** Ritcha Saxena

**Affiliations:** 1 University of Minnesota, Duluth, Minnesota, USA

The article provides 12 general recommendations for planning physician education. It emphasizes the importance of lifelong learning in the medical field and the necessity for physicians to stay updated with advancements in medicine and technology. Here are a few suggestions that could significantly enhance the quality of the paper.

- Consider including a section discussing the importance of considering accessibility and inclusivity when deciding on educational formats. Guidance on how to accommodate diverse learning needs, including those of different age groups and learning preferences can be helpful to ensure maximum engagement and effectiveness of the educational activities.  
- Also, in Tip 7, it could be beneficial to provide specific examples or case studies illustrating the selection criteria for speakers. This would help readers apply the recommendations effectively in real-world scenarios  
- It might also help to emphasize the importance of selecting speakers who not only possess expertise in the subject matter but also demonstrate effective teaching skills. If possible, examples of criteria for evaluating potential instructors can be described in a table, such as their ability to engage learners, to convey complex information clearly, and to facilitate interactive learning experiences.

**Grammatical errors: **It is advisable to proofread the article to ensure grammatical accuracy. A few errors are mentioned here –

- Tip 4: The repetition of the word "address" should be corrected.
- Tip 11: The goal of continuing education to close the professional gap. -> This should be - The goal of continuing education is to close the professional gap.
- It is an essential to measure change in physician knowledge, competence, performance, and patient outcomes. -> This should be: It is essential to measure change in physician knowledge, competence, performance, and patient outcomes.
- There are several ways to measure change,; pre- and post- tests and subjective evaluations and feedback are a couple... -> Remove the semicolon after "change"

Thank you for the opportunity to review.

If evidence from practice is presented, are all the underlying source data available to ensure full reproducibility?

Not applicable

Is the topic of the practical tips discussed accurately in the context of the current literature

Yes

Are all factual statements correct and adequately supported by citations?

Partly

Are the conclusions drawn balanced and justified on the basis of the presented arguments?

Yes

Are arguments sufficiently supported by evidence from the published literature and/or the authors’ practice?

Partly

Reviewer Expertise:

Medical Education, AI, Technology-enhanced Education

I confirm that I have read this submission and believe that I have an appropriate level of expertise to confirm that it is of an acceptable scientific standard, however I have significant reservations, as outlined above.

# Response

**Published:** 2024-04-22

**Authors:** Lisa Fore-Arcand

**Affiliations:** CME & Psychiatry-Behavioral Sciences, Eastern Virginia Medical School, Norfolk, Virginia, USA

Thank you for reviewing and providing detailed feedback on our paper.  We addressed your suggestions below in our revised paper.

1. We added content to Tip 8 - Assessing potential barriers, to address accessibility and inclusivity.
2. In Tip 7 we added content highlighting the importance of selecting speakers.
3. Regarding grammatical edits – we have address all.

# Reviewer report: Reviewer response for version 1

**DOI:** 10.21956/mep.21317.r36114

**Published:** 2024-03-25

**Authors:** Ghaith Al-Eyd

**Affiliations:** 1 Nova Southeastern University, Fort Lauderdale, Florida, USA

Thank you for the opportunity to review this interesting manuscript. In this article, the authors highlight the importance of continuing medical education (CME) as a vital lifelong learning activity for physicians and other health professionals. The authors designed a 12-step process to plan, develop, deliver, and evaluate a CME program. T

 he introduction section briefly addressed the role of CME to enhance physician skills and improve patient outcomes. However, it would be more effective if the introduction also addressed, using relevant cited references, the importance of nurturing lifelong learning skills across the medical education continuum (undergraduate and graduate) as a base for a successful future CME.

 Although it is explained in the introduction that the proposed 12 steps do not need to happen in a specific order, a logical sequence could be still needed to ensure a smooth flow of the design and to avoid overlaps across different steps.  Currently, there are some overlaps in the sub steps within some tips that makes some steps appear to be repetitive or need to be merged, e.g. designing the gap analysis for physician education (Tip 1) and then another step includes defining the target audience (Tip 3). Also, it’s not clear if the “planning Committee” (Tip 2) would also have a role in designing the gap analysis (Tip 1). Similarly, identifying the activity learning objectives was addressed in the first tip (Tip 1); those identified objectives would be more relevant if they were crafted based on the related core competencies (Tip 5). Furthermore, a CEM activity that targets, in addition to physicians, other health professionals, should address core competencies that are essential for the non-physician audience as well.

 In order to measure the effectiveness of the CME program (Tip 11), the identified outcome measures for the CME activity should evaluate whether the program objectives/outcomes have been achieved, and how well.

 It would be more effective if the manuscript also includes a “discussion” section that addresses the authors’ experience leading to the proposed 12 tips. The discussion could include examples of delivered CME activities and their related outcome evaluation results as well as related challenges and limitations and approaches used to address them.

 Please consider the followings clarifications and suggestions:

**Tip 1:** Grammatical editing is needed for “Once the problem is identified, determine the ideal performance and/or outcome is”. Suggested edit: “once the professional practice gap is identified, the ideal performance related to that gap needs to be determined next”?

**Tip 2:**

- Specify that the identified speakers are “experienced in the field of the selected CME topic”.
- Guided by the principles of adult learning, this committee will also identify the best ways to attract the target audience to attend the educational activity.

**Tip 4:** Consider the working/lifestyle and availability of the target audience when identifying the program delivery method that best ensures maximum participation.

**Tip 8:** Plan to “address” potential barriers

**Tip 9:** To clarify that the CME has no financial return goal, please consider editing “financial goal” to “determine the projected total expenses of organizing and delivering the activity” or any other similar clarification.

**Tip 11:** Typo (comma and semicolon) in “change,;”

**Limitations and Conclusion: **

- Please consider citing some references for the reported example challenges, here are some examples:

- Reis T, Faria I, Serra H, Xavier M. Barriers and facilitators to implementing a continuing medical education intervention in a primary health care setting. BMC Health Serv Res. 2022;22(1):638. Published 2022 May 13. doi:10.1186/s12913-022-08019-w
- O’Brien Pott, M., Blanshan, A.S., Huneke, K.M. et al. Barriers to identifying and obtaining CME: a national survey of physicians, nurse practitioners and physician assistants. BMC Med Educ 21, 168 (2021). https://doi.org/10.1186/s12909-021-02595-x
- Add “continuing” to “quality medical education” so it will be” quality continuing medical education”.
- The statement that describes the CME topic areas includes areas that are commonly identified for CME. That statement could be edited, e.g. “The future planning for CME usually addresses common areas related to state and national guidelines, pharmaceutical updates, and quality and safety topics such as initiatives and best practices. However, ongoing CME planning might also address emerging gaps and needs identified by central health authorities and organizations as well as by need analysis surveys”. 

Thank you again for this opportunity to review this manuscript that addresses an important topic on the lifelong learning required for physicians and other health care professionals.

If evidence from practice is presented, are all the underlying source data available to ensure full reproducibility?

Not applicable

Is the topic of the practical tips discussed accurately in the context of the current literature

Partly

Are all factual statements correct and adequately supported by citations?

Partly

Are the conclusions drawn balanced and justified on the basis of the presented arguments?

Partly

Are arguments sufficiently supported by evidence from the published literature and/or the authors’ practice?

Partly

Reviewer Expertise:

Medical Education, Pathology

I confirm that I have read this submission and believe that I have an appropriate level of expertise to confirm that it is of an acceptable scientific standard, however I have significant reservations, as outlined above.

# Response

**Published:** 2024-04-22

**Authors:** Lisa Fore-Arcand

**Affiliations:** CME & Psychiatry-Behavioral Sciences, Eastern Virginia Medical School, Norfolk, Virginia, USA

Thank you for your detailed feedback and time in reviewing our manuscript. We have incorporated the following changes, in our revised paper, based on your suggestions.

1. Based on your suggestion to add information on the importance of lifelong learning we added a sentence to the abstract. The abstract cannot include references.
2. Tip 11 – we reworded Tip 11 to address if the learning objectives and outcomes were achieved and how well.
3. We added brief content in the Introduction highlighting our years of experience.
4. Regarding grammatical edits – we have address all.
5. Tip 7 - addressed selecting expert speakers in the first sentence.
6. Tip 10 - highlights marketing and promotion of the activity.
7. Tip 4 - covers addressing selecting the best teaching methods while considering all factors.
8. Tip 8 - we changed plan ‘for’ to plan ‘to address’ protentional barriers based on your comments.
9. Tip 9 - we made minor adjustments to the paragraph. We did not change ‘financial goal’ because this describes all types of budgets.
10. Limitations and conclusions: we added ‘continuing’ to quality medical education and added a reference to support this section.

# Reviewer report: Reviewer response for version 1

**DOI:** 10.21956/mep.21317.r36113

**Published:** 2024-03-25

**Authors:** Rie Raffing

**Affiliations:** 1 The Parker Institute, Bispebjerg & Frederiksberg Hospital,, University of Copenhagen, Copenhagen, Denmark

This manuscript presents 12 tips for developing Physician Education and aims to provide support for designing education for physicians both in relation to the recent medical care standards as well ac clinical performance. 

 These 12 tips are all relevant and important to consider and take into practical application when developing a medical education curriculum for physicians. 

 Several of the tips are discussed in context of current and relevant literature, however some tips are unreferenced. It would strengthen the manuscript to do so. For example, "Curriculum Development for Medical Education" edited by Patricia A. Thomas et.al. could be relevant. 

 I believe it would strengthen Tip 3 to include a targeted needs assessment, after defining the target audience, in order to make sure to cover the needs of the future course participants.

 I believe it would strengthen Tip 12 to include feedback from the learners to the teachers, for example regarding communication skills, student involvement and subject knowledge. 

 Lastly, in cases where it is possible, I believe it is important to evaluate if the training programme result in the targeted outcomes in a clinical context. For example if the older people with HIV experiences a difference as a result  of the educational activity described in Table 1. Please see "The Kirkpatrick model" for inspiration (level 4). 

 Thank you for well written and easy-to-go-to tips for developing Physician Education.

If evidence from practice is presented, are all the underlying source data available to ensure full reproducibility?

Not applicable

Is the topic of the practical tips discussed accurately in the context of the current literature

Partly

Are all factual statements correct and adequately supported by citations?

No

Are the conclusions drawn balanced and justified on the basis of the presented arguments?

Yes

Are arguments sufficiently supported by evidence from the published literature and/or the authors’ practice?

Partly

Reviewer Expertise:

Medical education

I confirm that I have read this submission and believe that I have an appropriate level of expertise to confirm that it is of an acceptable scientific standard, however I have significant reservations, as outlined above.

## References

[1] : 12 tips for developing Physician Education. *MedEdPublish* .2024;14: 10.12688/mep.19901.1 10.12688/mep.19901.1 PMC1094084538495780

# Response

**Published:** 2024-04-22

**Authors:** Lisa Fore-Arcand

**Affiliations:** CME & Psychiatry-Behavioral Sciences, Eastern Virginia Medical School, Norfolk, Virginia, USA

Thank you for taking time to review and provide detailed comments on our manuscript. We appreciate your time. We have incorporated the following changes in our revised paper, based on your suggestions.

1. We added references to most tips.
2. The last sentence of the introduction states that these tips do not have to occur in this specific order. We address the needs assessment in Tip 1 – Design the gap analysis.
3. Based on your comment to provide feedback to the teachers we added it to Tip 11 - Evaluate and Change.

# Reviewer report: Reviewer response for version 1

**DOI:** 10.21956/mep.21317.r35834

**Published:** 2024-03-14

**Authors:** Peirce W. Johnston

**Affiliations:** 1 University of Cincinnati College of Medicine, Cincinnati, USA

Drs. McCoy and Fore–Arcand have proposed tips for educators to improve medical education. Their 12 tips itemize and describe articulate & well-reasoned steps for medical educators to "stay abreast of [healthcare] changes......to enhance their skills, knowledge, and performance."  

 The strength of these 12 tips lie in their capacity to assist physician educators, either in a stepwise fashion (1 through 12) or else to draw upon only those tips which apply to their project. Each tip discusses an important subject within the goals of physician education: designing the "gap analysis" (Tip 1); identifying the requisite "core competencies (Tip 5); examining financial needs (Tip 9);  and quantifying/measuring change (Tip 11). 

 Drs. McCoy and Fore–Arcand have supported their proposed tips with pertinent literature. The paragraph on Limitations wisely recognizes barriers to physician education, including "time constraints, cost, industry, influence, impact assessment, and limited interactivity," impediments that physician educators face on a daily basis, project to project. This paragraph to me represents a clear-eyed recognition of barriers to limitations that will help physician educators build more robust projects. 

 I don't have any specific advice for how to improve upon Drs. McCoy 's and Fore–Arcand's paper. I feel it answers questions existing within physician education and helps to guide physician educators with practical advice and examples.  Their Table 1, for instance, is an excellent means for outlining plans for an educational activity.   I applaud its succinct and eminently practical format.  I wish Drs. McCoy and Fore–Arcand the best and would be happy to answer any questions my peer review raises. Thank you for this opportunity, Dr. Peirce Johnston, MD

If evidence from practice is presented, are all the underlying source data available to ensure full reproducibility?

Not applicable

Is the topic of the practical tips discussed accurately in the context of the current literature

Yes

Are all factual statements correct and adequately supported by citations?

Yes

Are the conclusions drawn balanced and justified on the basis of the presented arguments?

Yes

Are arguments sufficiently supported by evidence from the published literature and/or the authors’ practice?

Yes

Reviewer Expertise:

Limited researcher but my areas are in Medical Student Education and PsychOncology

I confirm that I have read this submission and believe that I have an appropriate level of expertise to confirm that it is of an acceptable scientific standard.

# Response

**Published:** 2024-04-22

**Authors:** Lisa Fore-Arcand

**Affiliations:** CME & Psychiatry-Behavioral Sciences, Eastern Virginia Medical School, Norfolk, Virginia, USA

Thank for you time and positive feedback.

# Reviewer report: Reviewer response for version 1

**DOI:** 10.21956/mep.21317.r36112

**Published:** 2024-03-05

**Authors:** Anne M. Messman

**Affiliations:** 1 Department of Emergency Medicine, University Health Center-6G, Wayne State University School of Medicine, Detroit, MI, USA

This article presents 12 general recommendations aimed at those that would be planning a continuing education session for physicians. The tips are general and somewhat helpful, although seem targeted towards those that would be planning a larger educational intervention, specifically one with funding, not really for an intervention that would be performed on a smaller scale. It would be helpful if the title and abstract reflected this. Specific thoughts/recommendations are below:

 Tip 1 - table is helpful.

 Tip 4 - a table with examples of different teaching methodologies would be helpful.

 Tip 7 - grammatical error in second sentence (should say "Choose a speaker..." rather than "Choose speaker...").

 Tip 8 - an overlooked area that the authors should address is ensure that the activity is accessible to all learners, including those with disabilities.

 Tip 11 - multiple grammatical errors (first sentence should say "The goal of continuing education is to..." rather than "The goal of continuing education to..."; second sentence should say "It is essential..." rather than "It is an essential..."; third sentence has both a comma and a semicolon next to each other, need to get rid of comma).

If evidence from practice is presented, are all the underlying source data available to ensure full reproducibility?

Not applicable

Is the topic of the practical tips discussed accurately in the context of the current literature

Yes

Are all factual statements correct and adequately supported by citations?

Yes

Are the conclusions drawn balanced and justified on the basis of the presented arguments?

Yes

Are arguments sufficiently supported by evidence from the published literature and/or the authors’ practice?

Yes

Reviewer Expertise:

medical education

I confirm that I have read this submission and believe that I have an appropriate level of expertise to confirm that it is of an acceptable scientific standard, however I have significant reservations, as outlined above.

# Response

**Published:** 2024-04-22

**Authors:** Lisa Fore-Arcand

**Affiliations:** CME & Psychiatry-Behavioral Sciences, Eastern Virginia Medical School, Norfolk, Virginia, USA

Thank you for your thoughtful review of our manuscript. We have incorporated the following changes, in our revised paper, based on your suggestions. 

1. Regarding your suggestion to specify ‘what type of education we are describing’ we added to the title to reflect educational conferences.
2. We did not add a table with examples as recommend, but edited tip 4 to provide two examples of teaching formats when designing educational conferences.
3. Regarding grammatical edits – we have addressed all.
4. On Tip 8 we included content regarding accessibility.

# Reviewer report: Reviewer response for version 1

**DOI:** 10.21956/mep.21317.r35836

**Published:** 2024-02-16

**Authors:** Anita Kablinger, Alexandra Toloczko

**Affiliations:** 1 Department of Psychiatry and Behavioral Medicine, Carilion Clinic, Roanoke, Virginia, USA; 2 Virginia Tech Carilion School of Medicine, Roanoke, VA, USA

McCoy and Fore-Arcand provide 12 general tips for developing physician education. These tips are easy to read and important to consider when approached with developing physician education. To make the paper stronger, the reviewers recommend identifying the goal of the paper and the specified audience with greater detail. The paper "outlines a step-by-step plan for designing high-quality education programs for physicians", but in what context? Academic, private, community programs? All specialties? The audience appears to be physicians, but the introduction includes "other health care providers".  The tips are practical yet not specific. Tip 1 should describe "SMART" objectives with the appropriate references. Table 1 provides a good example of a planning tool, but the learning objectives are vague and, thus, the desired results are vague and are unable to be quantified. One of the greatest difficulties with CME education and implementation is the ability to confirm that the knowledge was received, and that the physician behavior reflects the new information. Describing methods to assess the backend of educational initiatives for knowledge implementation into healthcare, would be beneficial. Updated references would be welcome for each tip.

 Grammatical:

 Please revise this sentence within Tip 1 for grammar and flow:  "Once the problem is identified, determine the ideal performance and/or outcome is."

 Tip 7: "Choose speaker **s** based on the specifically determined criteria that will fulfill the educational needs of the target audience."

 Tip 11: "The goal of continuing education **is** to close the professional gap."

 Although Table 1 is a good addition to this practical brief paper, the reviewers recommend an example of a full Tip 1-12 idea for the reader's benefit.

 Thank you for allowing us to review this manuscript.

If evidence from practice is presented, are all the underlying source data available to ensure full reproducibility?

Not applicable

Is the topic of the practical tips discussed accurately in the context of the current literature

Partly

Are all factual statements correct and adequately supported by citations?

Partly

Are the conclusions drawn balanced and justified on the basis of the presented arguments?

Partly

Are arguments sufficiently supported by evidence from the published literature and/or the authors’ practice?

Partly

Reviewer Expertise:

Medical and research education.

We confirm that we have read this submission and believe that we have an appropriate level of expertise to confirm that it is of an acceptable scientific standard, however we have significant reservations, as outlined above.

# Response

**Published:** 2024-04-22

**Authors:** Lisa Fore-Arcand

**Affiliations:** CME & Psychiatry-Behavioral Sciences, Eastern Virginia Medical School, Norfolk, Virginia, USA

We really appreciate you taking time to review and provide detailed feedback. Following are the changes we made, in our revised paper, based on your feedback:

1. For your suggestion to ‘identify the goal to the paper and specify audience with greater detail’ we updated the last paragraph of the abstract to strengthen the goal of the paper and specify the audience.

-This paper outlines a step-by-step plan for designing high-quality education programs for practicing physicians in any field. It aims to assist in developing ongoing education, aligning physician practices with the latest medical care standards, and optimizing their clinical performance to improve patient and community health. -

1. ‘Other healthcare providers’ was removed from the introduction.
2. Regarding the learning objectives, we addressed designing SMART objectives.
3. We updated most tips to include references.
4. Regarding grammatical edits – we have address all.
5. Regarding your suggestion to develop a table illustrating tips 1-12, we respectfully decline because of the brief nature of this paper and time constraints.
